# Resistive Multi-Gas Sensor for Simultaneously Measuring the Oxygen Stoichiometry (*λ*) and the NO*_x_* Concentration in Exhausts: Engine Tests under Dynamic Conditions

**DOI:** 10.3390/s23125612

**Published:** 2023-06-15

**Authors:** Carsten Steiner, Thomas Wöhrl, Monika Steiner, Jaroslaw Kita, Andreas Müller, Hessam Eisazadeh, Ralf Moos, Gunter Hagen

**Affiliations:** 1 Department of Functional Materials, University of Bayreuth, 95440 Bayreuth, Germany; 2 CPK Automotive GmbH & Co. KG, 48157 Münster, Germany

**Keywords:** emissions, oxygen stoichiometry, nitrogen oxides, gas sensor, engine testing, exhaust gas, high temperature, characterization, thick-film technology, thin-film technology, multi-layer, sensor concept

## Abstract

Due to increasingly stringent limits for NO_*x*_ emissions, there is now more interest than ever in cost-effective, precise, and durable exhaust gas sensor technology for combustion processes. This study presents a novel multi-gas sensor with resistive sensing principles for the determination of oxygen stoichiometry and NO*_x_* concentration in the exhaust gas of a diesel engine (OM 651). A screen-printed porous KMnO_4_/La-Al_2_O_3_ film is used as the NO_*x*_ sensitive film, while a dense ceramic BFAT (BaFe_0.74_Ta_0.25_Al_0.01_O_3–δ_) film prepared by the PAD method is used for λ-measurement in real exhaust gas. The latter is also used to correct the O_2_ cross-sensitivity of the NO_*x*_ sensitive film. This study presents results under dynamic conditions during an NEDC (new European driving cycle) based on a prior characterization of the sensor films in an isolated sensor chamber with static engine operation. The low-cost sensor is analyzed in a wide operation field and its potential for real exhaust gas applications is evaluated. The results are promising and, all in all, comparable with established, but usually more expensive, exhaust gas sensors.

## 1. Introduction

Nitrogen oxide (NO*_x_*) emissions are strictly limited worldwide due to their toxic effects on both humans and the environment. Combustion engines play a key role in this regard and appropriate sensor technology is required for effective strategies to suppress NO*_x_* formation [1,2,3]. Most of the deployed sensors from large-scale production are based on ceramic multi-layer technology with oxygen ion conducting YSZ ceramics [2,4]. The complex electrochemical sensors use multiple cells to first measure oxygen stoichiometry and NO*_x_* concentration separately in a second chamber. The sensor elements are produced in HTCC (high-temperature co-fired ceramics technology) and are still expensive from an economical perspective; furthermore, they operate with small signal amplitudes (current in the nA/ppm NO_*x*_) for the detection of NO*_x_* concentration [5,6]. Cross-sensitivities to other gases, such as ammonia (NH_3_), are also typical [7]. Cheaper alternatives to the existing sensors are therefore desirable.

Based on these considerations, initial studies focusing on alternative NO*_x_* sensors have been presented recently, using a resistive sensing principle with a film of potassium permanganate (KMnO_4_) that could be implemented fairly inexpensively [8,9,10,11]. Previous studies have observed a decreasing electrical resistance, with an increasing NO*_x_* concentration. The impedimetric change of the sensor material was attributed to the sorption of NO*_x_* on KMnO_4_ (nitrate formation) [9]. At high temperatures, the electrical resistivity of the material was determined by the balance of sorption and desorption, which was also affected by the *p*_O2_ in the environment [9,10,12,13]. A higher *p*_O2_ favored nitrate formation and simultaneously affected both the base resistance and the NO*_x_* sensitivity of the sensor film [9,14]. Under real exhaust gas conditions, knowledge of the exact oxygen concentration, or oxygen stoichiometry *λ*, in the exhaust gas is therefore essential for evaluating the NO*_x_* concentration *c*_NO_*x*__.

To address this problem, the signal of the KMnO_4_ film was combined with the information from an oxygen sensor in previous studies [15]. The first measurements using a commercial wideband lambda sensor (UEGO sensor, universal exhaust gas oxygen sensor) could already show that this concept could be used to estimate the NO*_x_* concentration in the real exhaust of a diesel engine [16]. The approach allowed basically to assess the NO*_x_* emissions; however, significant differences in the determined *c*_NO_*x*__ could be found in parts compared with the common NO*_x_* sensor technology. The most probable reason for this was the fact that the sensitivities of the KMnO_4_ film (with respect to NO*_x_* and O_2_) had been only performed in laboratory conditions and a detailed characterization of the film properties in real exhaust gas was lacking. As an additional consequence, the specification of a proper strategy to correct the oxygen influence was challenging. Furthermore, two individual sensors, one for *c*_NO_*x*__ and another for *λ* measurement, were required.

Building upon these ideas, an extended sensor concept was presented in recent studies that combined the NO*_x_* sensitive KMnO_4_ film with an O_2_ sensitive film on the same sensor substrate [14,15]. The so-called multi-gas sensor thus offered capabilities equivalent to those of modern NO_*x*_ sensors, namely the simultaneous determination of *c*_NO_*x*__ and *λ*. Additionally, in this case, the concept was based on ceramic planar technology using resistive sensor principles for both sensor films. For the O_2_ sensitive film, metal oxides (that defect chemically interact with the *p*_O2_ in the environment) were particularly suitable at elevated temperatures. The electrical properties of some of these materials were even independent of temperature, as is commonly known from SrTi_1–*x*_Fe*_x_*O_3–*δ*_ (STF) [17,18,19], Co_1–*x*_Mg*_x_*O_1–*δ*_ [20], and SrTi_1–*x*_Mg*_x_*O_3–*δ*_ [21]. However, for materials such as STF, the issue of the irreversible inactivation by sulfurization in exhaust applications has not yet been solved [17,18,19,22]. In this study, a material from the barium iron tantalate (BFT) family was chosen as the O_2_ sensitive film. In the correct composition (stoichiometric tantalate content 25%), it also showed temperature independent sensor characteristics above 600 °C [23,24,25,26]. Its resistive response to the ambient oxygen concentration at high *p*_O2_ (>10^–4^ bar) was based on the formation of holes $h^{•}$ by filling oxygen vacancies $V_{O}^{••}$ in the lattice. Under these conditions, BFT typically behaved as a *p*-type conductor with a decreasing impedance at higher oxygen concentrations [25]:

(1) $$\frac{1}{2}O_{2}+V_{O}^{••}↔O_{O}^{x}+{2h}^{•}$$

The material has been widely studied in laboratory experiments and defect chemistry models for high and low *p*_O2_ have also been published [23,24,25,26,27,28]. A recent study also investigated for the first time the sensor response in lean-burn diesel engine exhaust [29]. The results were encouraging and consistent with the findings on the oxygen exchange mechanisms in the material, making BFT a promising candidate for the application as a resistive O_2_ sensitive film in the multi-gas sensor, even if the *p*-type conducting behavior of BFT limits the application to lean conditions, such as in diesel exhausts.

Initial investigations were also performed on the combined sensor setup (multi-gas sensor), but were limited to measurements in synthetic gas mixtures that simulate real exhausts [14,15]. Therefore, the aim of this study was to analyze the sensor concept under dynamic conditions in the exhaust gas of a diesel engine. For this purpose, the (cross-) sensitivities of the individual films (KMnO_4_, BFT) in real exhaust gas were analyzed in advance. From the determined characteristics, the oxygen stoichiometry and the NO*_x_* concentration were monitored in an NEDC (new European driving cycle) with the multi-gas sensor. In this process, the signal from the BFT film would also be taken to correct the O_2_ cross-sensitivity of the KMnO_4_ film. By direct comparison with commercial exhaust gas sensors, the potential of the multi-gas sensor with respect to the measurement of *λ* and *c*_NO_*x*__ in real exhaust would be discussed. The presented work therefore focused primarily on application-related aspects of the sensor concept but would also link the observed sensor reaction to its scientific background. Nevertheless, the isolated chemical mechanisms have been enlightened in more detail in other studies, to which we would like to refer at this point for KMnO_4_ [8,9,10,11,12,13] and BFAT [23,24,25,26,27,28,29].

Furthermore, the investigation also serves as a fundament to integrate further sensitive films to detect additional components in the exhaust gas, since the integration of other sensitive films on the sensor substrate is technically simple.

## 2. Materials and Methods

In this section, the experimental approach and the scientific fundamentals are explained. This comprises the introduction of the sensor element, the preparation of the sensitive films, and the description of the measurement setup at the engine.

### 2.1. Sensor Element

**Figure 1:**
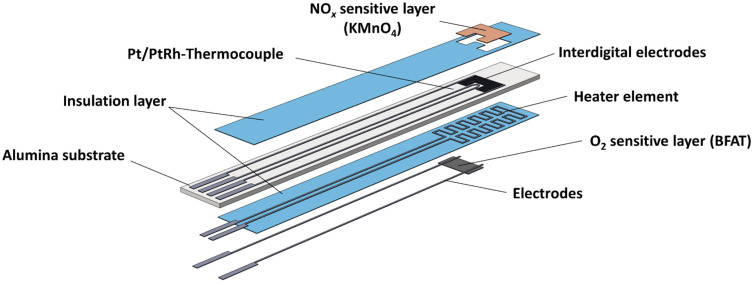
Design of the multi-gas sensor with porous NO_*x*_ sensitive and dense ceramic O_2_ sensitive film.

Figure 1 schematically shows the multi-layer structure of the sensor. The sensor consisted of the aluminum oxide substrate with screen-printed Pt/PtRh thermocouple (DuPont) and Pt interdigital (IDE) electrodes (Heraeus LPA 88-11S) on the front side. The porous NO*_x_* sensitive KMnO_4_ film, which was prepared by screen-printing, was located on top of the IDE structure and the rest of the substrate was covered by an overlying insulation layer (QM42). With the buried Pt heater structure (Heraeus LPA 88-11S) on the reverse side between the substrate and another insulation layer, the sensor operating temperature at the thermocouple was controlled to 600 °C. As a final component, two additional electrodes (Heraeus LPA 88-11S) were attached to the insulation layer on the reverse side for resistive measurement of the O_2_ sensitive BFAT film (barium iron aluminum tantalate, composition: BaFe_0.74_Ta_0.25_Al_0.01_O_3–_*_δ_*). The dense nanocrystalline BFAT film was produced via powder aerosol deposition (PAD). Details of the ceramic PAD process were given in [30], whereas the preparation of the sensor was similar, as described in [24].

**Figure 2:**
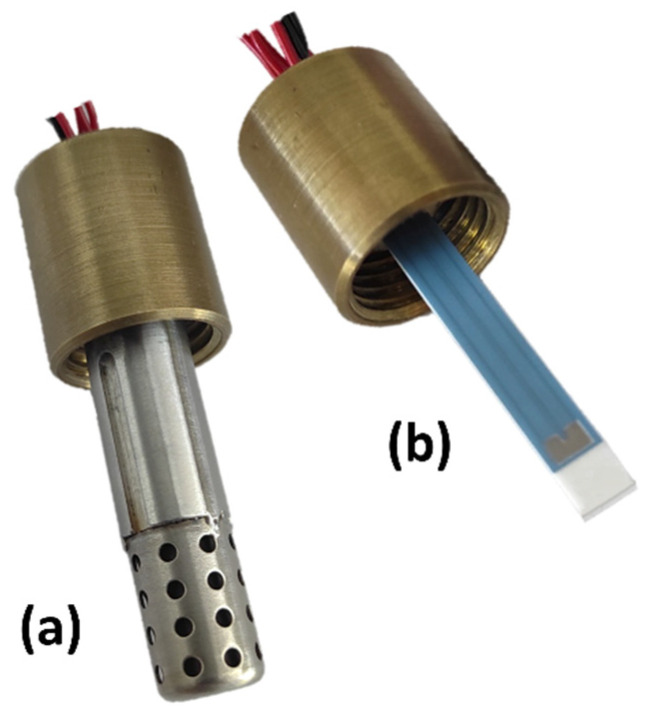
Multi-gas sensor element integrated in the housing (**a**) with and (**b**) without the protective cap.

For characterization in the exhaust gas, the sensor element was integrated into a housing with a protective cap, which is also presented in Figure 2. The complete sensor with the protective cap that was used in the engine experiments is illustrated in Figure 2a. Additionally, Figure 2b shows the sensor element integrated in the housing.

### 2.2. NO_x_ Sensitive Film

The porous NO*_x_* sensitive film of potassium permanganate (KMnO_4_) on a lanthanum-stabilized *γ*-alumina (3% La) was prepared via the “incipient wet impregnation” method, similar to [8,9,31,32]. In this process, KMnO_4_ was dissolved in water and applied to a *γ*-Al_2_O_3_ powder in a multi-step process. The prepared powder was converted into a screen-printable paste, from which a porous thick film (thickness about 30 µm) was prepared. The NO*_x_* concentration was determined using the resistive sensor principle on the interdigital electrode (IDE) structure (100 µm × 100 µm) by 2-wire impedance spectroscopy at 40 kHz (PalmSens4), with an applied effective voltage of 0.1 V. For better comparability, the relative change Δ*R*_KMnO4,rel_ of the film resistance was evaluated in the following.

### 2.3. O_2_ Sensitive Film

The powder for the BFAT film was prepared via the mixed oxide route, analogously to [28,33,34]. BaCO_3_ (Alfa Aesar, 99%), Fe_2_O_3_ (Alfa Aesar, 98%), Al_2_O_3_ (Almatis, 99.8%), and Ta_2_O_5_ (Alfa Aesar, 99%) powders were weighed stoichiometrically and homogenized in a planetary ball mill (Fritsch Pulverisette 5, Idar-Oberstein, Germany) in ZrO_2_ milling jars (stabilization: 3.5% MgO) with ZrO_2_ milling balls (Ø 10 mm, stabilization: 5.0% Y_2_O_3_) at 180 rpm for 4 h in cyclohexane The total batch size was 25 g. Subsequently, the powder was dried at 200 °C for at least one day and then sieved (90 µm mesh) to remove large agglomerates. Calcination was carried out in a chamber furnace at 1350 °C for 15 h. The powder was then homogenized again. Then, the calcined powder was ground again in two stages to reduce the particle size ZrO_2_ grinding balls (5.0% Y_2_O_3_) of size Ø 20 mm (1 h) and Ø 10 mm (3 h), respectively. The grinding medium was again cyclohexane. Finally, the powder was sieved again (90 µm mesh). The BFAT powder was then deposited onto the sensor element with masking, using the PAD method. Here, O_2_ with a volume flow of 8 L/min was used as the carrier gas for the aerosol generation. The nozzle (spacing to substrate 2 mm) was moved at a velocity of 5 mm/s with 150 sweeps. The dense ceramic BFAT films were approximately 5 µm thick. X-ray diffraction (XRD) patterns und scanning electron microscope (SEM) images can be found for example in [29]. The typical nanocrystalline film consisted of grains with a size of tens of nm. The DC resistance of the BFAT film (2-wire) was measured with a digital multi-meter (Keithley 2700 series). Again, the relative change Δ*R*_BFAT,rel_ of the resistive BFAT film signal was evaluated.

### 2.4. Experimental Setup

**Figure 3:**
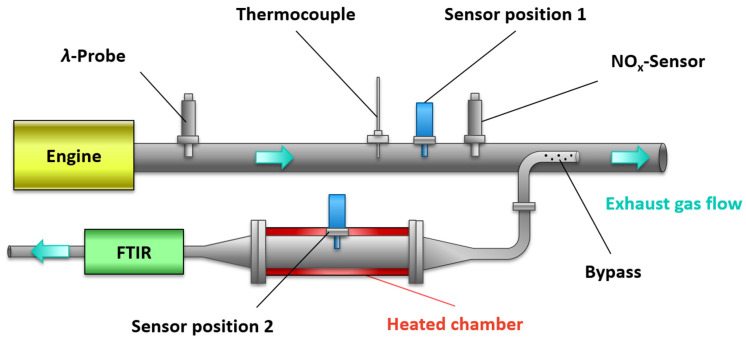
Schematic layout of the experimental system with sensor position 1 in the exhaust pipe and position 2 in a bypass with thermally insulated chamber, which was kept constant at 300 °C. The sensor operation temperature is 600 °C.

In this study, the sensor was investigated in the exhaust gas of a Mercedes-Benz OM 651 (4-cylinder in-line engine, engine displacement 2.1 L, compression ratio 16.2:1, 150 kW). The engine had an exhaust gas turbocharger and was operated without exhaust gas recirculation (EGR) to achieve larger *c*_NO_*x*__ levels. The measurement setup is shown schematically in Figure 3. Various sensors were available for chemical exhaust gas analysis. A wideband lambda probe (UEGO, BOSCH LSF 4.9) close to the engine was used to monitor the oxygen stoichiometry *λ*, which was defined as:

(2) $$\lambda =\frac{\mathrm{supplied}\;\mathrm{air}\;\mathrm{volume}}{\mathrm{stoichiometrically}\;\mathrm{required}\;\mathrm{air}\;\mathrm{volume}},$$

The UEGO sensor also served as a control probe of the ECU (electrical control unit) for correct engine operation. The multi-gas sensor was operated in two different positions. Position 1 was located directly in the exhaust pipe (standard tube Ø 60 mm) and was exposed to the total exhaust flow of the engine. Close to sensor position 1, a commercial NO*_x_* sensor (Continental UniNO*_x_*) was installed to monitor the NO*_x_* concentration in the exhaust gas, in addition to a type *K* thermocouple to measure the exhaust gas temperature. In addition, the total exhaust gas mass flow was logged by the measurement system of the engine test bench.

Before the sensors were operated in full stream, a bypass measurement (sensor position 2) was performed to characterize the sensor response and the response of the gas sensitive layers, respectively. A sample with a constant volume flow (approx. 5 L/min) was extracted via a pump through a sampling tube (Ø 10 mm) with small holes, which was oriented in the flow direction of the exhaust gas. In contrast to sensor position 1, the bypass measurement with a temperature-controlled sensor chamber (300 °C, Figure 3, red) allowed the sensor element to be examined at constant operation temperatures and gas flow conditions. Therefore, the response to the chemical gas composition could be determined here free from thermal influences and flow effects. In addition to the gas sensors, an FTIR (MultiGas 2030 FTIR Gas Analyzer, MKS Instruments, Andover, MA, USA) was connected to the setup to analyze the extracted sample flow in the bypass.

## 3. Results and Discussion

This section is divided by subheadings. It provides a concise and precise description of the experimental results, their interpretations, as well as the experimental conclusions that can be drawn.

### 3.1. Sensor Characterization

For the sensor characterization (calibration curves), the signal in the bypass measurement (Figure 3, position 2) was investigated at constant operating conditions in the exhaust gas. While the determination of the sensitivity to oxygen was sufficient for the BFAT film, both the NO*_x_* sensitivity and the cross-sensitivity to oxygen stoichiometry (*λ*) had to be analyzed for the KMnO_4_ film. For this purpose, different operating parameters were set on the engine, covering an oxygen stoichiometry of about 1.65 < *λ* < 6.75 and NO*_x_* concentrations of about 100 ppm < *c*_NO_*x*__ < 1050 ppm. Both variables (*λ*, *c*_NO_*x*__) were varied independently from each other to separate their contributions. Different oxygen stoichiometries *λ* were adjusted by changing the boost pressure *p*_Boost_. The NO*_x_* concentration could be modified by specifically adjusting the injection angle *α*_injection_ with the ECU.

**Figure 4:**
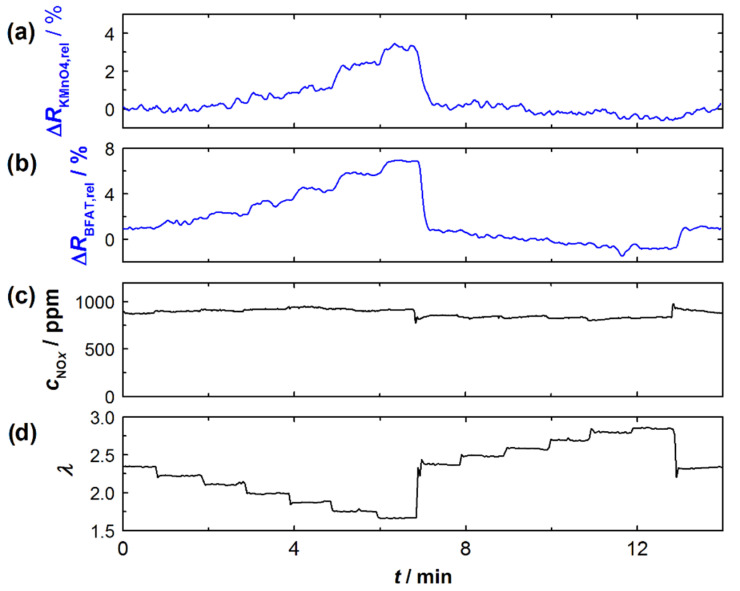
Experiment to determine the effect of oxygen stoichiometry *λ* on the signals (relative change in resistance) of the two sensor films: (**a**) relative resistance change of the KMnO_4_ film, (**b**) relative resistance change of the BFAT film, (**c**) NO*_x_* concentration according to the commercial NO*_x_* sensor, and (**d**) oxygen stoichiometry according to the commercial UEGO sensor.

Figure 4 exemplarily shows an experiment on the effect of oxygen stoichiometry *λ* in the exhaust gas. At the top (Figure 4a), the relative signal amplitudes of the KMnO_4_ and BFAT films (blue) are shown. Below that, in Figure 4b, *c*_NO_*x*__ and *λ* are listed (black), each measured with the NO*_x_* sensor and the UEGO lambda probe close to the engine (as sketched in Figure 3). While *λ* was changed stepwise, the NO*_x_* concentration in the exhaust gas remained (almost) unchanged. As can be seen, the resistances of both sensor films responded to changes in *λ* in the exhaust gas. Moreover, it could be observed that the changes increased noticeably with lower *λ*. This behavior was also previously observed for the BFAT film in [14], although in a smaller operating window. Experiments similar to those shown in Figure 4 were performed at different operating parameters in order to obtain representative data for a wider operating window of the sensor. In addition, the experiments on the *c*_NO_*x*__ dependence of the KMnO_4_ were performed. As expected, the signal from the BFAT film showed no response to the changes in *c*_NO_*x*__ during these experiments.

**Figure 5:**
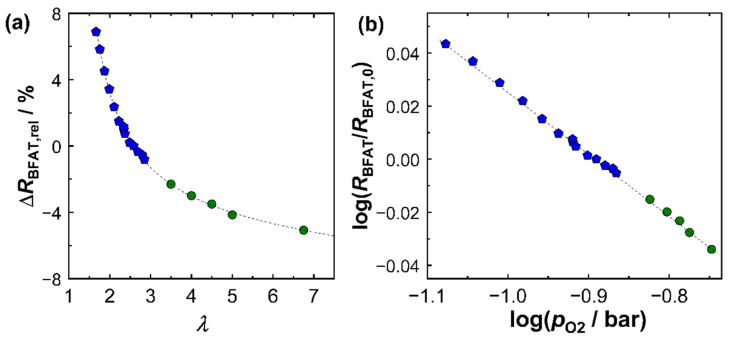
Signal of BFAT film in exhaust gas: (**a**) effect of the oxygen stoichiometry *λ* on relative signal amplitude (sensor response) Δ*R*_BFAT,rel_ and (**b**) resistance change as a function of *p*_O2_ (logarithmic scale). Blue data are derived from Figure 4. Green data mark further operation points.

Based on this series of investigations, a superordinate calibration for the sensitivities of both sensor films considering multiple operation modes is presented as follows. First, Figure 5 shows the signal of the BFAT film as a function of the oxygen concentration in the exhaust gas. Figure 5a shows the relative change in resistivity (Δ*R*_BFAT,rel_) versus oxygen stoichiometry *λ*. The change in signal amplitude was based on the BFAT resistance value at *λ* = 2.50. The blue data points were derived directly from Figure 4. Points from other operating points are shown in green. In the wider operating window, the trend of a smaller sensitivity with a leaner exhaust gas composition was further confirmed. The plausible reason for this phenomenon was that, at large lambda values, the *p*_O2_ in the exhaust gas hardly changed and was close to the air concentration (21% O_2_).

If the change in resistance of the BFAT film was plotted against the *p*_O2_ (logarithmically), as in Figure 5b, a linear relationship was obtained. The dependence confirmed that the sensing mechanism followed expectations from the defect chemistry of the BFAT material, i.e., the formation of holes $h^{•}$ by filling oxygen vacancies. This was also confirmed by the slope in the resistance *R*_BFAT_ in Figure 5b, which was calculated to be −0.235. Considering that +1/4 was typically found for the BFAT conductivity σ (∝1/R), the primary p-type conduction character at high oxygen partial pressures was a plausible finding and was in line with previous studies [25,28].

From the same experiments, the *λ* cross-sensitivity of the KMnO_4_ film was determined, which is summarized in Figure 5a, again referring to *λ* = 2.50. At low lambda values (*λ* < 2.50), a significant influence of oxygen stoichiometry on the resistance of the KMnO_4_ film was observed. On the other hand, at high excess oxygen values (*λ* > 2.5), the influence could almost be neglected. Applied on engine operation, this result meant that the cross-sensitivities had to be taken into account, especially at high loads (high engine power), since these operating points were usually associated with a drop to lower *λ*. At operating points with high excess oxygen, on the other hand, such as idle engine phases, the NO_*x*_ sensor response remained largely unaffected by *λ*.

**Figure 6:**
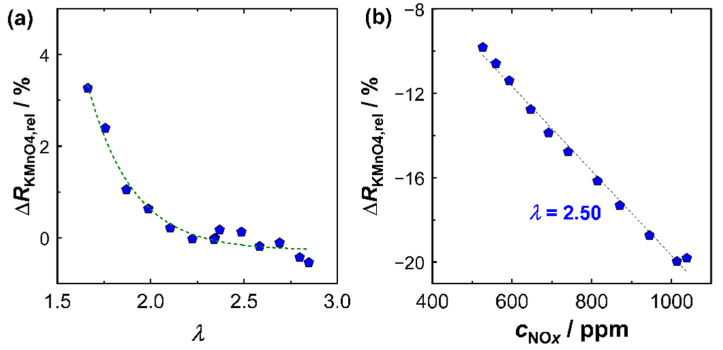
Signal of KMnO_4_ film in exhaust gas: (**a**) cross-sensitivity of relative signal amplitude (Δ*R*_KMnO_4_,rel_) to the oxygen stoichiometry λ and (**b**) Δ*R*_KMnO_4_,rel_ as a function of the NO_*x*_ concentration.

Last but not least, the NO*_x_* sensitivity of the KMnO_4_ film had to be determined. For this purpose, experiments with different NO*_x_* concentrations were performed in a similar manner. Figure 6b shows the result of the investigation at high NO*_x_* concentrations (525 ppm < *c*_NO_*x*__ < 1040 ppm) at a constant oxygen stoichiometry *λ* = 2.50. In the investigated operating window, there was a direct proportionality of the relative resistance change to *c*_NO_*x*__ in the exhaust gas. This correlation was also found in other operation modes. However, it was also observed that the sensitivity of the KMnO_4_ film $S_{{\mathrm{NO}}_{x}}^{{\mathrm{KMnO}}_{4}}$ was a function of the oxygen stoichiometry *λ* and confirmed findings from laboratory experiments of previous studies [14]. The sensitivity of the KMnO_4_ film $S_{{\mathrm{NO}}_{x}}^{{\mathrm{KMnO}}_{4}}$ was defined by Equation (3) as:

(3) $$S_{{\mathrm{NO}}_{x}}^{{\mathrm{KMnO}}_{4}}(\lambda )=-\frac{\Delta R_{{\mathrm{KMnO}}_{4},\mathrm{rel}}(\lambda )}{c_{{\mathrm{NO}}_{x}}},$$

In this study, a proportionality between NO*_x_* sensitivity and oxygen stoichiometry was assumed. The variables used to calculate the current NO*_x_* sensitivity were statistically determined from a series of different operating modes that could be used for an operating field between 1.65 < *λ* < 6.75 and *c*_NO*_x_*_ < 1050 ppm:

(4) $$S_{{\mathrm{NO}}_{x}}^{{\mathrm{KMnO}}_{4}}(\lambda )=-(3.33\cdot 10^{-3}\cdot \;\lambda_{\mathrm{BFAT}}+1.17\cdot 10^{-2})\frac{\%}{\mathrm{ppm}}$$

with the oxygen stoichiometry $\lambda_{\mathrm{BFAT}}$ in the exhaust gas determined by the response of the BFAT film. At the reference lambda value of *λ* = 2.5, the KMnO_4_-film had a NO*_x_* sensitivity of about 20%/(1000 ppm NO*_x_*), which was in line with Figure 6b. The NO*_x_* sensitivity never decreased below 15%/(1000 ppm NO*_x_*) for lean exhausts (*λ* > 1), but could rise by a factor of more than 1.5 for high oxygen stoichiometries (*λ* > 6). Equation (4) represents a simple approach to determine $S_{{\mathrm{NO}}_{x}}^{{\mathrm{KMnO}}_{4}}$. In laboratory studies [14,16], it was found that the sensitivity of KMnO_4_ depended not only on *λ* but also on *c*_NO_*x*__ itself, specifically that $S_{{\mathrm{NO}}_{x}}^{{\mathrm{KMnO}}_{4}}$ decreased with higher NO*_x_* concentrations. According to the former results, the correlation shown in Figure 6b was therefore expected to be nonlinear over a wider range of NO*_x_* concentrations, especially with a higher $S_{{\mathrm{NO}}_{x}}^{{\mathrm{KMnO}}_{4}}$ at low NO*_x_* concentrations. However, these conditions were technically not accessible due to restrictions in the ECU to guarantee a proper engine operation. Furthermore, during engine operation a correlation between *λ* and *c*_NO_*x*__ was observed: during periods of low load (idle phases), high lambda values (*λ* > 5) were typical. At the same time, *c*_NO_*x*__ was small (<200 ppm) due to low combustion temperatures. Following the previous findings, both high *λ* and low *c*_NO_*x*__ thus favored a high $S_{{\mathrm{NO}}_{x}}^{{\mathrm{KMnO}}_{4}}$ for these exhaust gas compositions. Conversely, lower *λ* (<3) and high *c*_NO_*x*__ (>500 ppm) appeared, particularly at high engine loads, which reduced $S_{{\mathrm{NO}}_{x}}^{{\mathrm{KMnO}}_{4}}$ in those cases. This trend is included in Equation (3) by the calibration measurements. Equation (3) therefore probably does not represent the isolated effect of *λ* on $S_{{\mathrm{NO}}_{x}}^{{\mathrm{KMnO}}_{4}}$, but rather considers the combined effect of oxygen stoichiometry *λ* and NO*_x_* concentration. During multiple experiments at the engine, $S_{{\mathrm{NO}}_{x}}^{{\mathrm{KMnO}}_{4}}$ was observed to decrease by about one third due to the contribution of both effects. A more extensive investigation of the separate contributions of *λ* and *c*_NO_*x*__ in real exhaust gas would be interesting from a research standpoint, but, as stated above, was hardly possible due to the limited range individual operating parameters that were allowed in the ECU. In addition, they would provide only a minor benefit for the application itself, since a correlation between both contributions could be observed anyway. The chosen approach was therefore a simple and effective method, but still applied only to the engine operating window investigated in this study.

### 3.2. Performance during Dynamic Engine Operation

**Figure 7:**
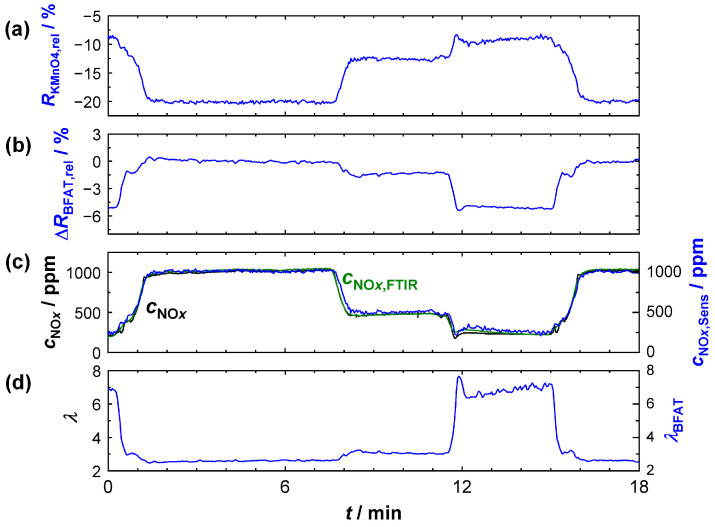
Sensor signal when measured in the bypass with variation of the engine operation points: (**a**) relative resistance change of the KMnO_4_ film; (**b**) relative resistance change of the BFAT film; (**c**) NO*_x_* concentration according to the commercial NO*_x_* sensor (black), the FTIR (green), and calculated from the KMnO_4_ resistance (blue); (**d**) oxygen stoichiometry according to the commercial UEGO sensor (black) and calculated from the BFAT resistance (blue).

First, it was investigated whether the above characterization of the sensitive films could be used to correctly determine the oxygen stoichiometry and the NO*_x_* concentration when measured in the bypass (sensor position 2). The sensor operation temperature was again 600 °C. For this purpose, initial tests were conducted on the engine test bench with operating point variations, which are shown in Figure 7. Specifically, operating points representing a wide range of different *λ* and *c*_NO_*x*__ were chosen here. Figure 7a,b are plots of the relative resistance changes (Δ*R*_KMnO4,rel_, Δ*R*_BFAT,rel_) of the KMnO_4_ and the BFAT film (blue), respectively. In Figure 7c,d, *c*_NO_*x*__ and *λ* in the exhaust gas are presented, again measured with the NO*_x_* sensor and the UEGO lambda probe (black). In addition, *c*_NO_*x*__ was also recorded with the FITR (green). In blue, the calculated curves from the signals of the two sensitive films (*λ*_BFAT_, *c*_NO_*x*_,Sens_) are shown. The oxygen stoichiometry *λ*_BFAT_ in Figure 7d was determined accordingly from the data of the BFAT film. The calculation of *c*_NO_*x*_,Sens_ was carried out from the combined information of both sensitive films (BFAT and KMnO_4_) and thus included the corrections for the *λ*-dependent resistance and the NO*_x_* sensitivity $S_{{\mathrm{NO}}_{x}}^{{\mathrm{KMnO}}_{4}}$ of the KMnO_4_ film.

The derived *λ* signal (measured and calculated as described above) obtained from the BFAT film coincided with the signal of the commercial UEGO lambda probe and reliably reproduced the real conditions in the exhaust gas. The *λ* signal from the BFAT film (Figure 7d) was stable and accurate, especially at low lambda values, which was plausible based on the increasing *p*_O2_ sensitivity (Figure 5a,b). At high *λ*, the calculated oxygen stoichiometry exhibited a larger noise. Since the oxygen concentration hardly changed here due to the already high *p*_O2_ in the exhaust and, consequently, the expected signal amplitude of the BFAT film was lower, interferences played a more crucial role in this case. However, it should also be mentioned at this point that even the output of the commercial UEGO lambda probes could differ strongly under these conditions. The reliability of the exact values must therefore be critically rated for both sensors.

**Figure 8:**
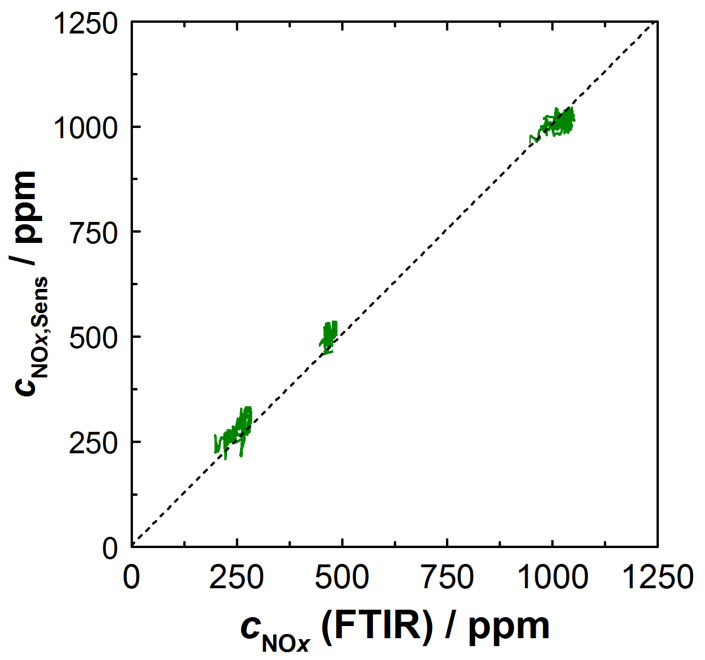
Calculated NO*_x_* concentration *c*_NO_*x*_,Sens_ over *c*_NO*_x_*_ measured with FTIR.

The derived NO*_x_* signal *c*_NO_*x*_,Sens_ (measured and calculated as described above) obtained from the NO_*x*_ sensitive film also coincided with the NO*_x_* concentration *c_NO_*_x_ measured with the FTIR (Figure 7c). The approach with *λ*-correction of the resistance and the NO*_x_* sensitivity provided plausible results in the entire operating window, which were equally confirmed by the commercial NO*_x_* sensor and by the FTIR data. Our experience here showed that significantly better results were obtained, especially when correcting the KMnO_4_ sensitivity. As expected, the oxygen cross-sensitivity of the KMnO_4_ film was mainly required in the range of λ < 2.5. Similar to the calculated lambda *λ*_BFAT_, operating points with low *c*_NO_*x*__ and high lambda (12 min < *t* < 15 min) were more affected by perturbations. An analysis of the calculation showed that these perturbations were mainly directly due to signal fluctuations in the KMnO_4_ film, which originated from the high sensitivity $S_{{\mathrm{NO}}_{x}}^{{\mathrm{KMnO}}_{4}}$ at these operating points. Instead, error propagation from the *λ*-correction was largely avoided with the BFAT film since O_2_ cross-sensitivity played a small role under these conditions. Nevertheless, the data showed that both films responded rapidly and that the oxygen stoichiometry and NO*_x_* concentration could be resolved in time during dynamic transitions to a different operating point. The NO*_x_* concentration could be reliably calculated from the resistance signals of the two films; this is also shown in Figure 8. There, the calculated concentration *c*_NO_*x*_,Sens_ was plotted against the NO*_x_* concentration measured by FTIR. Shown are the steady-state operating points from the experiment in Figure 7. The measured data are along the angle bisector and confirm that almost identical values for the NO*_x_* concentration were obtained with both measurement systems.

**Figure 9:**
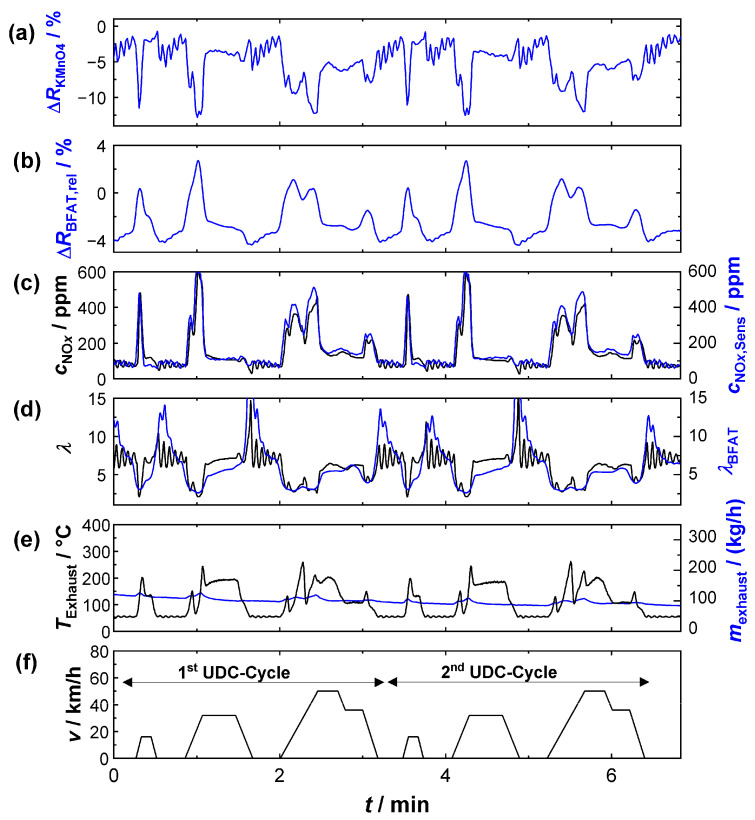
Sensor signals during two UDCs: (**a**) relative signal amplitude of the KMnO_4_ film; (**b**) the BFAT film; (**c**) NO_*x*_ concentration according to the commercial NO_*x*_ sensor (black) and calculated from the KMnO_4_ signal (blue); (**d**) oxygen stoichiometry according to the UEGO lambda probe (black) and calculated from the BFAT signal (blue); (**e**) exhaust gas temperature (black), exhaust gas mass flow (blue), and (**f**) vehicle speed.

Once the calibration was successfully applied to measurements in the bypass, the following section presents a series of experiments that were carried out with the sensor in the full exhaust gas stream (sensor position 1). An NEDC was established on the engine test bench to represent dynamic driving conditions. The official test cycle consisted of an urban driving cycle (UDC) (executed four times with low engine power and acceleration) and an extra-urban driving cycle (EUDC), which simulated driving at higher speeds. The results of two of these UDCs are shown in Figure 8. The top two plots again contain the relative resistance signals of the KMnO_4_ (Figure 9a) and of the BFAT (Figure 9b) films. Below that, in Figure 9c,d, the signals (*λ*, *c*_NO_*x*__) from the conventional exhaust gas sensor are again shown in black and the signals derived from the multi-gas sensor (*λ*_BFAT_, *c*_NO_*x*_,Sens_) are shown in blue. The FTIR data were not included this time, since the sometimes very dynamic conditions could not be resolved due to the limited time resolution of the FTIR device. The exhaust gas mass flow data (black) from the engine controller and the exhaust gas temperature (blue) near the probe are also shown (Figure 9e). The bottom graph, Figure 9f, shows the velocity during the NEDC.

The segment shown in Figure 9 comprises two urban cycles. Each cycle in turn comprised several acceleration phases with partial load operation, in which lambda values in the range of 2 < *λ* < 5 were typical (e.g., *t* = 2.3 min). During the short acceleration phases, the exhaust gas mass flow (Figure 9e) temporarily increased and oxygen stoichiometry *λ* drops (Figure 9d), and peaks in the NO_*x*_ concentration (Figure 9c) were simultaneously observed. For both exhaust gas properties (*λ*, *c*_NO_*x*__), the multi-gas sensor provided similar values to the commercial sensors during these operating phases. Even NO*_x_* peaks were detected and the concentrations were also quantitatively similar (peaks up to 500 ppm), despite the highly dynamic conditions during the acceleration phases.

Between the phases of partial-load operation there were sections where the engine was mainly idling at low power (e.g., *t* = 5 min). In these phases, the ECU obviously set the oxygen stoichiometry to a higher oscillating lambda value. The *λ*-oscillation was also detected at the BFAT film. The amplitude of the resistance change was quantitatively small due to the small *p*_O2_ changes under these conditions but sufficiently high to derive a plausible lambda value. However, the calculated curve could only partially follow the oscillation frequency due to the limited timely resolution of the resistivity measurement (<0.7 Hz) at the BFAT film. In addition, it should not be ignored that the sensor positions of the UEGO lambda probe (close to the engine) and the multi-gas sensor were different and thus different local oxygen stoichiometries could be present at the sensors due to diffusion. Although the evidence could not be provided here, we assumed, based on the flanks of the resistivity signal, that the film itself responded faster to the oxygen stoichiometry in the exhaust gas than could be detected during data acquisition.

Oscillations were also observed for *c*_NO_*x*__ during idle engine phases. The concentrations here were small and were below 200 ppm. Again, identical *c*_NO_*x*__, and even similar oscillation amplitudes, could be measured with the multi-gas sensor, which was obviously a benefit from the higher sensitivity $S_{{\mathrm{NO}}_{x}}^{{\mathrm{KMnO}}_{4}}$ under these conditions. Thus, the measurement also showed that the oscillating oxygen stoichiometry hardly affected the NO*_x_* measurement, but the achievable accuracy at these operating points mainly depended on the signal quality of the KMnO_4_ film. In summary, the determination of exhaust gas characteristics with the multi-gas sensor succeeded in partial-load engine modes. The results of the resistive sensor principle were comparable with those of the established sensor technology.

**Figure 10:**
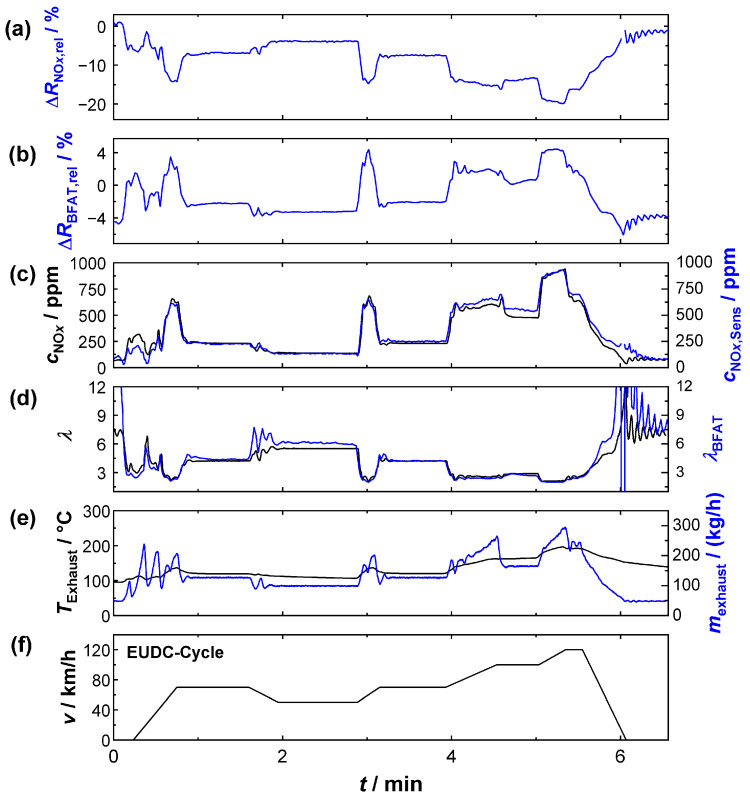
Sensor signals during EUDC: (**a**) relative signal amplitude of the KMnO_4_ film, (**b**) the BFAT film; (**c**) NO*_x_* concentration according to NO*_x_* sensor (black) and calculated from the KMnO_4_ signal (blue); (**d**) oxygen stoichiometry according to UEGO lambda probe (black) and calculated from the BFAT signal (blue); (**e**) exhaust gas temperature (black), exhaust gas mass flow (blue), and (**f**) vehicle speed.

Based on the findings from the UDC, the results of the extra-urban cycle (EUDC) are shown in Figure 10. The signals listed and the assignments of colors are identical to Figure 9. Unlike the UDC, the average engine power was higher to simulate driving outside of urban areas; the exhaust gas mass flows and the temperatures were therefore higher. At the same time, idling phases hardly played a role. For the exhaust gas composition, this meant a lower (average) oxygen stoichiometry and higher NO*_x_* raw emissions (peaks > 900 ppm). In this case, too, the determination of *λ* and *c*_NO_*x*__ worked successfully with the multi-gas sensor. Despite the more dynamic conditions during the acceleration phases, all flanks were recorded and the exhaust gas properties could also be quantified. Unlike in the UDC, the oxygen cross-sensitivity of the KMnO_4_ film also played a more important role in this cycle due to the temporarily lower *λ*-values. This effect could be corrected in the calculation by the oxygen information obtained from the BFAT film, which also showed higher signal amplitudes under these conditions.

Figure 9e and Figure 10e also show that flow conditions and exhaust gas temperatures were by no means constant during the test. The results from the NEDC cycle (UDC, EUDC) were all the more surprising, because such effects at the sensor were not considered in this study. On closer inspection (e.g., Figure 10e), the measurements showed that deviations between the multi-gas sensor and the commercially available probes were more likely to occur during rapid changes in the engine operation mode. An evaluation, which, e.g., also considered the heating power of the sensor, could probably improve the quality of the measurement even more. Nevertheless, the experiments showed that even the simplified method was sufficient to determine plausible results for the resistive multi-gas sensor.

Finally, the accuracy of the multi-gas sensor was quantified and compared with the specifications of commercial sensors and probes. For this purpose, it made sense to look at the KMnO_4_ and BFAT layers separately, starting with the latter. For the oxygen sensitive film, similar observations could be made as for commercial wideband probes: the sensor accuracy was significantly higher at low lambda values due to the higher change in *p*_O2_. Towards leaner oxygen stoichiometries, the measurement accuracy decreased noticeably, since the *p*_O2_ hardly changed anymore. In this case, a uniform quantification of the measurement uncertainty for the entire measurement range was therefore not practicable. Following the example of commercial probes, we therefore decided to refer the accuracy to a specific lambda value (here, *λ* = 2.59). For the measurements at static conditions, a standard deviation of the sensor signal of ±0.038 was observed. Typical wideband sensors (such as Bosch LSU 4.9 [35]) showed ±0.05 according to their specifications at *λ* = 1.7, which was a similar level. In dynamic conditions, the deviations could be greater. However, in this case, a direct comparison between the two sensors was not recommended due to the different sensor positions in the exhaust pipe.

To determine *c*_NO_*x*__, the reference sensor (Continental UniNO*_x_*-Sensor [35]) had an accuracy of ±10% for *c*_NO_*x*__ > 100 ppm (the latter condition was met for almost all operating conditions investigated). In most phases, including the NEDC cycle, the multi-gas sensor and the reference sensor did not differ by more than ±25 ppm. Exceptions were again observed under highly dynamic conditions (short acceleration phases with high *c*_NO_*x*__). However, one should keep in mind the different sensor positions. Considering the ±10% accuracy of commercial sensors, we therefore assumed that the multi-gas sensor could provide similar accuracies. This was also supported by the analysis with the FTIR in Figure 8. Furthermore, commercial NO_*x*_ sensors typically came with their own electronics for sensor operation and evaluation. Compared with commercial standards the evaluation presented here was carried out with simple methods, mainly focused on the material effects. It is likely that more detailed analysis could improve the sensor’s potential even further in future studies.

## 4. Conclusions

The measurements with the multi-gas sensor showed that with the two sensitive films (KMnO_4_, BFAT) and a resistive sensor principle, the oxygen stoichiometry (*λ*) and the NO*_x_* concentration (*c*_NO_*x*__) in the exhaust gas could be determined. Due to the preceding characterization of the sensitive films, results comparable to the established exhaust gas sensor technology in a wide operating window could also be obtained under dynamic conditions during an NEDC cycle of a lean-burn engine. At the same time, the simple sensor design and measurement principle has the potential to provide cost benefits over the industrial standard sensor systems. The approach of correcting the O_2_ cross-sensitivity of the KMnO_4_ film with the information of the BFAT film offers promising results (although the sensing chemistry of the two materials is based on different sensor mechanisms). Due to the planar sensor technology, the integration of other functional films to determine additional exhaust gas components is also conceivable. Additionally, possible cross-sensitivities to *λ* and *c*_NO_*x*__, which are typical for many NH_3_-sensitive materials, could be corrected with information from the existing films (similar to this study). Overall, encouraging results were obtained with the sensor concept. For further improvement, analyses on thermal effects seem to be useful, for example, including the heating power. Moreover, studies on the long-term stability of the sensor films and resistance against poisoning or inactivation under operating conditions are required.

## Data Availability

All relevant data presented in the article are stored according to institutional requirements and as such are not available online. However, all data used in this paper can be made available upon request to the authors.
